# Feasibility and therapeutic efficacy of a two-week low-level laser acupuncture therapy for shoulder and neck pain in office workers: Protocol for a pilot, single-blind, double-armed, randomised controlled trial

**DOI:** 10.1371/journal.pone.0260846

**Published:** 2022-01-21

**Authors:** Carol Chunfeng Wang, Lisa Whitehead, Travis Cruickshank, Johnny Lo, Jianhong (Cecilia) Xia, Jun Wen

**Affiliations:** 1 School of Nursing and Midwifery, Edith Cowan University, Joondalup, Western Australia, Australia; 2 School of Medical and Health Sciences, Edith Cowan University, Joondalup, Western Australia, Australia; 3 School of Science, Edith Cowan University, Joondalup, Western Australia, Australia; 4 School of Earth and Planetary Sciences, Curtin University, Perth, Western Australia, Australia; 5 School of Business and Law, Edith Cowan University, Joondalup, Western Australia, Australia; Monash University, AUSTRALIA

## Abstract

**Background:**

Shoulder and neck pain (SNP) is common in office workers and represents a serious public health problem given its detrimental impact on quality of life, physical functioning, personal finances, employers, and the health care system. Management with painkillers has adverse implications such as tolerance, addiction, and opioid abuse. Safe, sustainable, cost-effective, and evidence-based solutions are urgently needed. The non-invasive, painless, non-infectious, and safe modality of low-level laser acupuncture (LLLA) has shown promise for SNP management.

**Objective:**

The overarching aim of this study is to provide evidence of the feasibility and therapeutic efficacy of LLLA for office workers with SNP.

**Methods:**

This is a pilot, single-blind, double-armed, randomised controlled trial on the feasibility and therapeutic efficacy of a two-week LLLA therapy for office workers with SNP, aged 18 to 65 years. Each of the two study groups will contain 35 participants: the intervention group will receive LLLA from a licensed acupuncturist at the researchers’ university clinic (10–20 min/session, 3 sessions/week) for two weeks; the control group will receive usual care without painkillers. Outcomes will be measured at baseline, throughout the two-week intervention, and at trial end. Surveys including open-ended questions will be completed. The primary outcome of this study is to evaluate the feasibility of a two-week LLLA therapy for office workers with SNP, as measured by recruitment and completion rates, patient safety, and treatment adherence and compliance. Participants’ attitudes, motivation, and challenges to participation, intervention non-compliance, and experience of participating in the trial will be investigated via qualitative data. The secondary outcome is to evaluate the therapeutic efficacy of LLLA on SNP using the visual analogue scale (VAS) and the Short-Form McGill Pain Questionnaire (SF-MPQ); the work productivity and activity assessment (WPAI:SHP); 12-Item Short Form Survey (SF-12) for quality of life assessment; and the past 3-month out-of-pocket (OOP) cost for prescription and non-prescription SNP therapy, which is an indicative of the economic burden of SNP on patients and health care systems. This study was approved by Edith Cowan University’s Human Research Ethics Committee (No. 2021-02225-WANG).

**Results:**

Data collection will commence in December 2021 with anticipated completion by December 2022.

**Conclusions:**

Safe, sustainable, cost-effective, evidence-based interventions are needed to minimise the negative implications of SNP in office workers. LLLA is a promising modality in managing SNP. However, more consolidated evidence is required to provide insight regarding the effectiveness of LLLA. This study is expected to contribute to the challenging work of reducing the burden of SNP in office workers.

**Trial registration:**

Australian New Zealand Clinical Trials Registry (ANZCTR): ACTRN12621000426886p; https://www.anzctr.org.au/ACTRN12621000426886p.aspx

## Introduction

Shoulder and neck pain (SNP) (pain is felt at the top of the shoulder and over the upper arm without the specific disease) is a common and burdensome condition in office workers [1, 2] and affect an estimated 42–63% prevalence worldwide [3, 4]. Symptoms of SNP include pain in the neck, shoulder, and/or upper chest; neck stiffness; and difficulty turning the head. SNP represents a serious public health problem considering its detrimental impact on quality of life, physical functioning, personal finances, employers, and the health care system [4–9].

Extended working hours on the computer, prolonged sitting, and static postures are the main contributing factors to SNP [10], and medication and physiotherapy are the most common intervention strategies for managing this condition [10]. To date, optimal condition management remain is unknown and current medication regimens involving painkillers (e.g., opioids, NSAID) carry adverse implications, such as developing tolerance, addiction, and opioid abuse.

Acupuncture has been the oldest therapeutic modality in TCM and gained tremendous popularity as a complementary and alternative treatment in the west, especially in the last 20 years [11]. There are three main effects acupuncture treatment aims to achieve: regulation of Yin and Yang (restoring the relative equilibrium), reinforcing the vital Qi (energy force) and eliminating pathogenic influences, and ensuring Qi and blood flow through the meridians (channels in the body) [11]. Studies have demonstrated that acupuncture therapy can serve as a promising treatment modality [12]; for instance, acupuncture is widely used in to manage pain by activating acupoints on the body, therefore activating acupoint’s special composition of blood vessels, mast cells, and nerve fibres that mediate the acupuncture signals, inhibit glial cell activation and inactive the spinal microglia and astrocytes, mediates the immediate and long-term analgesic effects [13].

Low-level laser therapy (LLLT), also known as cold laser or photobiomodulation, involves the application of specific wavelengths at low power density over the injured area. LLLT acts like an anaesthetic agent that temporally disrupts the cytoskeleton. The exact mechanism for this effect is unknown, but it has been shown that LLLT promote vasodilatation, enhance blood flow, lymph drainage and fibroblast and neutrophil activation, which results in changes in the pain threshold [14]. The modulation of neurotransmitters is another possible mechanism of pain relief, as serotonin and endorphin levels have been shown to increase following laser treatment of myofascial pain in patients [15]. Fast-acting pain relief occurs as a result of a neural blockade of the peripheral and sympathetic nerves and the release of neuromuscular contractions leading to a reduction of muscle spasms [16, 17]. This treatment method has been identified as a complementary option in SNP management to relieve pain and improve quality of life [18, 19]. LLLT has been shown to be safe and the therapy possesses analgesic [18, 19] and anti-inflammatory effects, improves blood circulation, boosts immunity and accelerates wound healing [20].

Low-level laser acupuncture (LLLA) is one of the more recent technological developments in acupuncture that integrates cutting-edge laser technology with a centuries old modality in traditional Chinese medicine [21]. Instead of needle stimulation on acupuncture points, low-intensity non-thermal laser irradiation is applied to elicit cellular level physiological effects with sufficient energy [22]. LLLA is non-invasive, painless, non-infectious, and safe to use [23]. This form of acupuncture has also become increasingly popular among patients with needle phobias, particularly older people and children [24, 25].

Several studies have documented LLLA as a promising modality in pain management [26, 27]. Over the last three years, five reviews favoured LLLT in pain management thanks to its safety, efficacy, and clinical effectiveness [28–32]. However, there is no consolidated evidence of LLLA use in SNP management in this special study population; more studies are needed to provide timely insight.

The overarching aim of this study is to provide evidence of the feasibility and therapeutic efficacy of LLLA for office workers with SNP.

## Materials and methods

### Approval and registration

This study was approved by the Human Research Ethics Committee at Edith Cowan University (ECU) (No. 2021-02225-WANG). The trial has been registered with the Australian New Zealand Clinical Trials Registry (ANZCTR): ACTRN12621000426886p. Issue Date: 16 April 2021. Protocol Amendment Number: 02. Author: Carol Wang.

Funding: in-kind support by ECU.

### Study design

This study will commence in December 2021 and is anticipated to be completed by December 2022. The study is a pilot, single-blind (assessors blind to group allocation) randomised controlled trial with two parallel arms—an intervention group and a control group—of office workers aged 18 to 65 years with SNP. The trial will be conducted in Western Australia in the acupuncture research lab at ECU. This location is easily accessible by bus or public transport.

**Fig 1:**
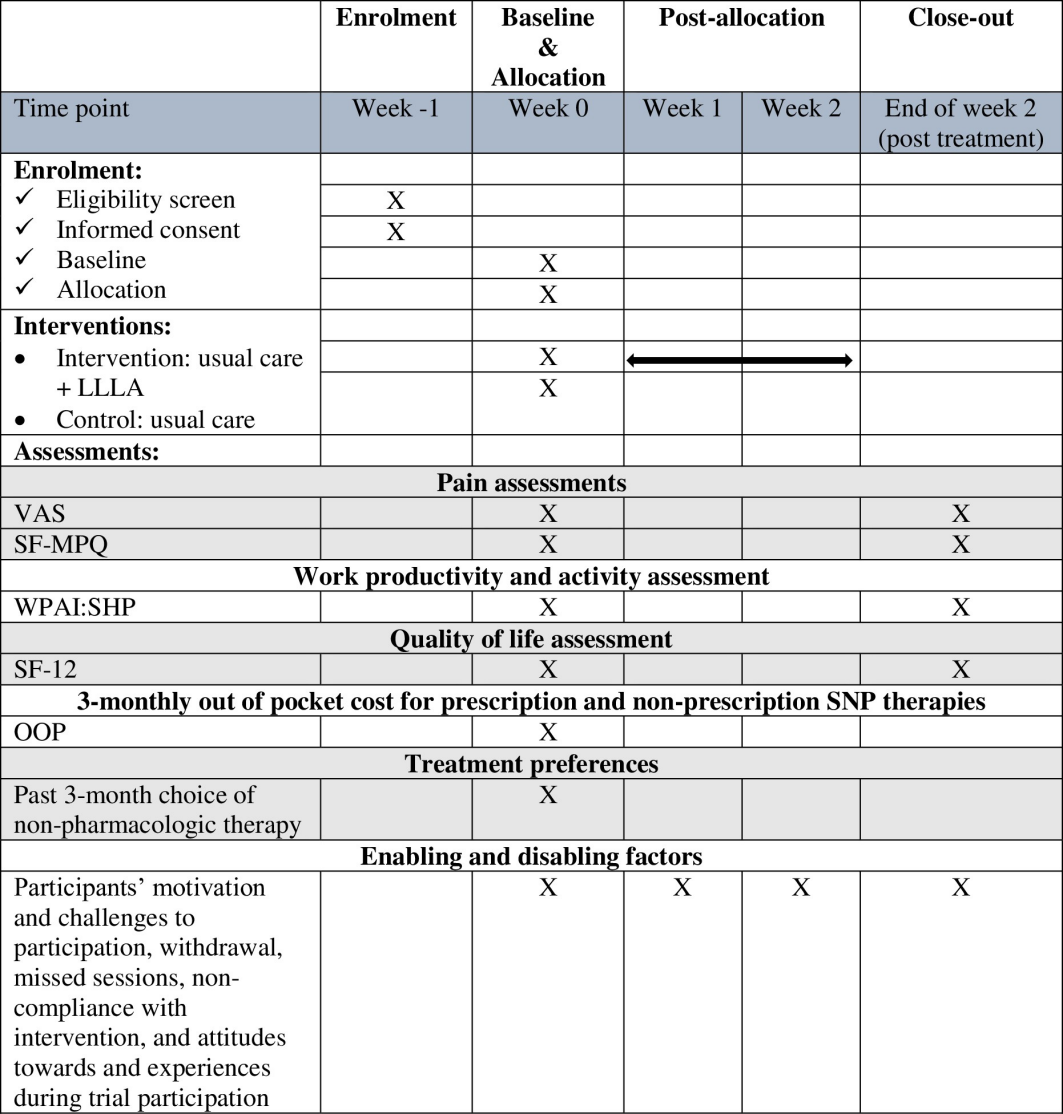
The schedule of enrolment, interventions, and assessments.

This protocol is conducted according to the Standard Protocol Items: Recommendations for Interventional Trials (SPIRIT) guidelines [33] (Fig 1). The intervention description follows the Template for Intervention Description and Replication (TIDieR) checklist and guide [34]. The study will be reported according to the Consolidated Standards of Reporting Trials (CONSORT) [35] guidelines and Consolidated Criteria for Reporting Qualitative Research (COREQ) [36].

#### Study population and eligibility criteria

As of June 2019, individuals aged between 15 and 65 years are referred to as the ’working-age population’ in Australia [37]. Seventy office workers will be recruited from the community for this trial. The participants will be aged 18–65 years, working more than 28 hours per week (< than full time as some people with SNP may work reduced hours) in sedentary office jobs, and have experienced moderate to severe SNP (VAS > 5/10 and SF-MPQ > 2/45) [38] for more than three months. People who have a severe health condition of the neck or shoulder (e.g., injury), have a fever, are highly sensitive to light, are diagnosed with cancer, or are pregnant will not be eligible.

#### Recruitment

Participants will be recruited via public advertisements, including through print, radio, and social media. Snowballing techniques will be applied to enhance recruitment. Individuals interested in participating in the study will be encouraged to contact the research team via email for an eligibility check. We will send interested respondents an online screening checklist, including the SF-MPQ and VAS, to assess their suitability for participation in accordance with our inclusion/exclusion criteria.

Our research team will contact people eligible to participate in the study by sending the first 70 eligible protentional participants (first come, first serve) with a participant information letter and a link (starting with a consent form) to complete an online survey once they have signed the consent form by ticking a box to confirm they agree to the conditions. The online survey should take no longer than 20 minutes to complete.

The 10 to 20-minute treatment sessions will occur outside working hours. As such, employer approval is not required. The intervention will be delivered across a range of days and times and participants will be expected to choose a session that does not conflict with their normal working hours.

### Randomisation

Following survey completion for the baseline measurements, the participants will be assigned to either the control or treatment group, with the assistance of a blinded statistician, via a randomised block design. This ensures the sample number in each group remain relatively similar through the recruitment process until the quota is reached.

1. The intervention group will receive six laser acupuncture treatment sessions from a licensed acupuncturist at ECU’s Acupuncture Research Clinic.
2. The control group will receive NO treatment but usual care (e.g., exercise) over the two-week trial

Participants in both groups will be de-identified and provided with a unique identifying number for future analysis.

### Sample size

As a pilot study, although it is feasible to recruit 30 participants per group [39], dropouts are likely during the trial process. We estimate 15% attrition based on the attrition of 12% reported in a previous study [40]. Taking these two factors into account, the sample size for this study will be 70, with 35 participants per study group to address feasibility issues (recruitment and completion rates, treatment adherence and compliance, and participants’ attitudes, motivation, and challenges to participation). The online questionnaires (hosted on Qualtrics) with a quantitative method and open-ended questions will assess the intervention and study design feasibility. It will inform future powered therapeutic effect trials for its outcome measures, treatment regime, and study design. Participants will be given a unique identification number, and the data collected will be treated with confidentiality and stored securely within the systems at the chief investigator’s university. Only authorised persons will have access to the collected data.

### Intervention

In addition to their usual care (e.g., exercise, painkillers), participants will receive LLLA from a licensed acupuncturist at ECU Acupuncture Research Clinic. Each session will last 10–20 minutes, including preparation, treatment, and conclusion of treatment, conducted three times weekly for two weeks. The 3B Laser Pen (200mW) with a spot area of 0.07cm^2^ used in the intervention will have a wavelength of 808 nm in continuous wave mode to be applied to bare skin. Each pressure point will receive 20 seconds of energy (4J/point), with 5 minutes being the maximum treatment time (60 J). According to TCM treatment of SNP, the acupuncture points (G SI 3 Houxi, SI 9 Jianzhen, SI 10 Naoshu, SI 11 Tianzong, B 21 Jianjing, LI 4 Hegu, LU7 Lieque, LI 11 Quchi, LI 14 Binao, LI 15 Jianyu, LI 16Jugu, TE 14 Jianliao, TE 15 Tianliao, GV 14 Dazhui, and ST 44 Neiting) were used on the most painful side. The acupuncture points were also chosen in consultation with clinician who is a licensed acupuncturist.

### Control

Participants in this group will be informed via email that they are in the no-treatment group (usual care only). As same as the intervention group, the control group will also be emailed an online link to complete the post-intervention questionnaires in two weeks’ time. Participates in this group will receive a complimentary LLLA after the two-week trial.

### Outcomes

**Fig 2:**
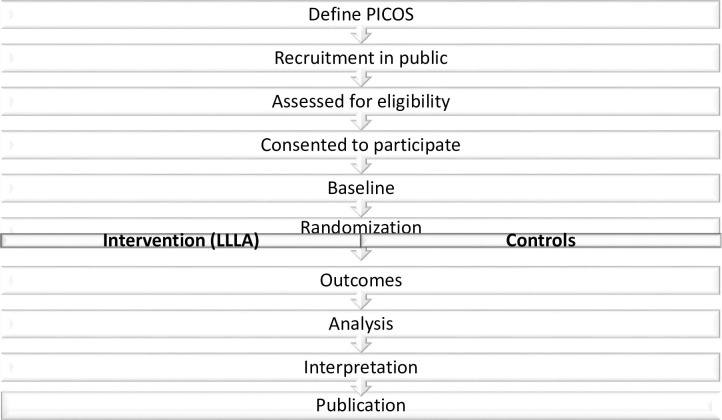
Flowchart of the protocol.

The primary outcome (feasibility) will be assessed throughout the study duration. Secondary outcomes will be measured at baseline (week 0) and following two weeks of LLLA including measures of pain, work productivity, and quality of life. Demographic data, the past 3-month OOP costs for prescription and non-prescription SNP therapies and questions on non-pharmacologic therapy will also be captured in the questionnaire. Measures are expected to take participants 50–60 minutes to complete in total (Table 1). If withdrawal occurs, we will still follow up with the participants if they consent. Data collected up to the time of withdrawal will be included in analyses unless the participant explicitly asks for it to be withdrawn. Fig 2 shows the process of the protocol.

**Table 1:** Expected time to complete the online measurement questionnaire.

Measure	Time to complete (minutes)	Baseline	2 weeks
**Pain assessments**			
VAS	1	✓	✓
SF-MPQ	3	✓	✓
**Work productivity and activity assessment**			
WPAI:SHP	5	✓	✓
**Quality of life assessment**			
SF-12	3	✓	✓
**3-monthly out of pocket cost for prescription and non-prescription SNP therapies**			
OOP	2	✓	
**Treatment preferences**			
Past 3-month choice of non-pharmacologic therapy	1	✓	
**Enabling and disabling factors**			
Participants’ motivation and challenges to participation, withdrawal, missed sessions, non-compliance with intervention, and attitudes towards and experiences during trial participation	20	✓	✓
**Total questionnaire/online assessment time (mins)**	**35**	**19**	**16**

#### Primary outcome

The primary outcome of this study is the feasibility of the two-week LLLA therapy for office workers with SNP. Feasibility measure includes (1) recruitment and completion rates (No. of referred, No. of eligible, No. of enrolled, No. of withdrawals, trial recruitment rate, and trial completion rate); (2) patient safety (No. and severity of adverse events); (3) treatment adherence (No. of completed sessions and missed sessions) and compliance; and (4) participants’ attitudes, motivation, and challenges to participation, reasons for withdrawal, missed sessions, and non-compliance with the intervention will be investigated via open-ended questions in the study-specific online survey at the end of the trial. Recruitment and completion rates will be assessed during the entire trial process. Patient safety, treatment adherence and compliance will be assessed during the interventions. Online surveys will be administered at baseline (week 0), post-two weeks intervention (end of week 2).

#### Secondary outcome

The secondary outcomes will include pain assessments as measured by mean scores on the visual analogue scale (VAS) and the Short-Form McGill Pain Questionnaire (SF-MPQ); work productivity and activity assessment (WPAI:SHP); and Quality of life assessment (SF-12). These outcomes will be measured using two online surveys: baseline (week 0) and post-intervention (end of week 2). Questions on participants’ non-pharmacologic therapy preferences, socioeconomic status, and the past 3-month out of pocket (OOP) expenses will be included and measured at week 0; participants’ experiences of participating in the trial will also be measured at the end of week 2.

#### Pain assessments

*VAS and SF-MPQ*. The visual analogue scale (VAS) and the Short-Form McGill Pain Questionnaire (SF-MPQ) are commonly used clinically to assess SNP [38]. VAS is a reliable, validated tool with adequate sensitivity [41, 42] that is often used to assess pain intensity. Pain is a subjective experience; therefore, it cannot simply be objectively measured but must also be assessed. Multi-dimensional assessment tools can evaluate multiple aspects of pain, such as sensation, mood and intensity. The McGill Pain Questionnaire (MPQ) is the most well-known and popular multi-dimensional pain assessment tool. However, with 78 pain descriptors, it is often clinically impractical. The Short-Form McGill Pain Questionnaire (SF-MPQ) was published in 1987, consisting of 15 pain descriptors: 11 that assess the sensory dimension of pain and four that assess the affective dimension of pain. Descriptors are rated on an intensity scale of none (= 0), mild (= 1), moderate (= 2), and severe (= 3). Three pain scores are derived from the sum of the intensity rank values of the chosen sensory, affective, and total descriptors [38]. This questionnaire is used to measure the quality (i.e., using words to describe the pain, such as ‘sharp’, ‘dull’, ‘stabbing’, ‘burning’, ‘crushing’, ‘throbbing’, ‘nauseating’, ‘shooting’, ‘twisting’ or ‘stretching’) as well as the intensity of pain [43–46].

#### Work productivity and activity assessment

*WPAI*:*SHP*. The Work Productivity and Activity Impairment Questionnaire for Specific Health Problem V2.0 (WPAI:SHP) [47, 48] will be used to examine changes in work productivity throughout the trial period. The WPAI:SHP is a 6-item questionnaire that evaluates self-reported productivity and activity during the past week. It includes subscales for absence from work (absenteeism), lost productivity while at work (presenteeism), overall work impairment, and the effects on non-work-related activities. Higher subscale value (0–100%) indicate greater work or activity impairment [47, 48].

#### Quality of life assessment

*SF-12*. The 12-item Short Form Health Survey (SF-12) will be used to examine changes in health related quality of life during the trial period. The SF-12 is a self-reported outcome measure assessing the impact of health on an individual’s everyday life and their quality of life [49, 50]. The SF-12 is a shortened version of the SF-36 but uses the same eight domains as the SF-36, including (1) Limitations in physical activities because of health problems; (2) Limitations in social activities because of physical or emotional problems; (3) Limitations in usual role activities because of physical health problems; (4) Bodily pain; (5) General mental health (psychological distress and well-being); (6) Limitations in usual role activities because of emotional problems; (7) Vitality (energy and fatigue); and (8) General health perceptions. The SF-12 and SF-36 possess similar validity [51–53]. Scores on these eight domains are aggregated to form two final components: physical and mental wellbeing scores.

#### Out of pocket cost and treatment preferences

*OOP*. The past 3-month OOP costs for prescription and non-prescription SNP therapies and questions on non-pharmacologic therapy will also be captured in the questionnaire.

#### Socioeconomic status

Information on socioeconomic status will be collected using a demographics questionnaire (e.g., education level, employment status, average working hours per week, personal annual income).

### Analyses

For the primary outcome, rates of recruitment (no. consented/eligible), completion (undertaken baseline and follow-up tests), adherence (participant’s completed sessions/no. of sessions), and adverse events (number and number per participant hour) will be calculated. The secondary outcome will be assessed following intention-to-treat principles. Linear mixed modelling will be conducted to assess changes in secondary outcomes throughout the study. This model allows for the inclusion of missing data in an intention-to-treat analysis without imputations (e.g., last-observation-carried-forward). If necessary, the analysis will be adjusted for baseline levels and potential confounding factors. Normality assumptions will be assessed using the Shapiro-Wilk test. Statistical significance will be set at an alpha level of 0.05. Corrections will be applied to all analysed outcomes to account for multiple comparisons. Appropriate effects sizes will be reported and interpreted. The qualitative data collected via open-ended questions across the two online surveys will be used to help explain or elaborate on the quantitative data.

### Ethics and dissemination

The study will be conducted in accordance with the National Statement and the Australian Code for the Responsible Conduct of Research, 2018 (the ‘Research Code’), and has been approved by the ECU’s Human Research Ethics Committee (No. 2021-02225-WANG). The information letter explains the study, including the purpose and procedures, the voluntary nature of participation, and the option to withdraw at any time. Participants are also guaranteed confidentiality and secured data storage. Any adverse events arising will be reported and managed by the instructors and the research team. Data will be securely stored in ECU’s security location, and no unauthorized persons will have access to the collected data.

## Discussion

SNP is a common and burdensome condition in office workers. Safe, sustainable, and cost-effective interventions are warranted to reduce the negative implications arising from this condition. LLLA is a promising modality but consolidated evidence is required to better understand its effectiveness. This study will provide updated knowledge of LLLA’s feasibility and effectiveness in the management of SNP among office workers aged 18 to 65.

This study will improve understanding of how to provide LLLA for managing SNP in office workers. The study will evaluate the feasibility of the intervention regime and methodological design. Currently, there is no such modality designed for this population with SNP. The findings from this study can add value to the evidence base about how to acceptably involve complementary medicine for office workers SNP. The evaluation will look at the use of LLLA in the context of increased computer work, and such trials are urgently needed. The findings can provide updated knowledge on the value of non-pharmacological interventions in alleviating the challenge of reducing the burden of pain management.

Considering the high pertinence of this topic, our study design aims to assess multiple relevant outcomes and the effectiveness of a feasible intervention in a clinical practice setting to improve practice and inform clinical and policy decisions. Our design can speed the pace and increase the efficiency/cost-effectiveness of clinical research and can make it more applicable to the ‘real world’ clinical settings.

It is worth mentioning that we have chosen a randomised design to account for confounding factors as much as possible; however, many factors can affect study outcomes. For example, the current study cannot blind the participants and the practitioner. The data analyser will be blinded throughout the trial to minimise reporting bias. A licensed acupuncturist will provide the LLLA therapy following a treatment protocol to ensure standardised delivery.

Placebo-controlled trials are not achievable for acupuncture studies due to no sham techniques developed capable of acting as placebo treatments. If a sham technique involves touch with pressure, it will inadvertently activate the body’s physiological response and the control technique’s pathway. Sham acupuncture techniques, therefore, should not be used in acupuncture related clinical trials [54]; instead, pragmatic trials that designed to answer a question about decision making in clinical care [55], where the control treatment can be an established standard therapy or a no-treatment group [54]. The sham procedure may be possible in laser acupuncture trials, but more evidence is required in this field.

This study’s methodological strengths are the randomised controlled trial design, pre-registration in a clinical trials registry, and qualitative data nested in a quantitative design. The small sample size and short intervention period are study limitations.

The results of this study will provide updated knowledge on the value of non-pharmacological interventions in alleviating the future challenge of reducing the burden of SNP for office workers. LLLA could be an example of a safe, sustainable, and cost-effective intervention with promise as a complementary modality.

## Results

Ethical approval was received in May 2021 and the data collection is planned to begin in December 2021. We expect to publish the first study results in mid-2022. Upon completing the trial, all participants will individually receive written results from their participants and providing them with an overview of the study status.

## Supporting information

S1 Checklist SPIRIT checklist with page reference numbers. (DOC)

S1 File Trial protocol for ethics application. (DOCX)
