# Supplementary material for: Feasibility and therapeutic efficacy of a two-week low-level laser acupuncture therapy for shoulder and neck pain in office workers: Protocol for a pilot, single-blind, double-armed, randomised controlled trial
Source: PLoS One. 2022 Jan 21;17(1):e0260846. doi: 10.1371/journal.pone.0260846 (PMC8782333; doi:10.1371/journal.pone.0260846)
Supplement: S1 File — (DOCX) [file pone.0260846.s002.docx]

**S2 Trial protocol for ethics application**

| Synopsis |  |
| --- | --- |
| **Name of the Funding Source:** TBC | **Protocol Number:** ACTRN12621000426886p |
| **Study Title:** Feasibility and therapeutic efficacy of a two-week low-level laser acupuncture therapy for shoulder and neck pain in office workers: a pilot, single-blind, double-armed, randomised controlled trial | |
| **Short Title:** LLLA therapy for shoulder and neck pain | |
| **Planned Study Sites:** Perth, Australia | |
| **Number of Participants:** Seventy office worker aged 18-65, working more than 28 h a week with sedentary office work and have moderate to severe SNP (VAS > 5/10 & SF-MPQ >2/45) for more than three months will be recruited for the study | |
| **Chief Investigator:**  Dr Carol Wang  School of Nursing and Midwifery  Edith Cowan University  Western Australia, 6027 | |
| **Study Period:** December 2021 – December 2022 | |
| **Objectives:**  The primary objective of this study is to evaluate the feasibility of two weeks low-level laser acupuncture (LLLA) for office workers with shoulder and neck pain (SNP).  The secondary objective is to evaluate the therapeutic efficacy of LLLA on pain, work productivity and activity, and quality of life in office workers with SNP. | |
| **Study Design:** This study is a single-blind (assessors blind to group allocation), double-armed, randomised controlled trial. | |
| **Study Criteria:** office workers with SNP will be recruited for this study.  Inclusion criteria:   1. Aged between 18-65 years 2. Working more than 28 h a week with sedentary office work 3. Moderate to severe SNP (VAS > 5/10 & SF-MPQ > 2/45) for more than three months   Exclusion Criteria:   1. With a serious health conditions of the neck and shoulder 2. Pregnant 3. With cancer 4. High sensitivity to light | |
| **Sample Size:** Based on previous LLLA therapy intervention studies, n = 35 participants will be required per group (total n=70) for this study. | |

# Investigators’ qualifications

Dr Carol Wang, the Chief Investigator, has 28 years working experience in the field of health. She worked in China, Singapore and Australia as a registered nurse in many areas, including mental health. She is also a licensed acupuncturist, which enables her to conduct clinical trials in Traditional Chinese Medicine (TCM) field. The diagnosis and treatment of TCM is fundamentally holistic, which always taking into account one's mental state and emotional wellbeing, as such, she has been working on a program of research that focuses on complementary and alternative therapy fits into the Australian Medical Research and Innovation Priorities, the ECU’s research themes and priority areas and the key research areas in the School of Nursing and Midwifery. As such, with the support of the university and her school, she has established the ECU Acupuncture Research Clinic and has several publications in the field of TCM.

Professor Lisa Whitehead is a leading nursing researcher in the field of improving clinical outcomes and working with families to support the management of chronic conditions. In her role as Professor of Nursing Research, Associate Dean Research, Director, Centre for Nursing, Midwifery and Health Services Research, Honorary Professor of Nursing Research, University of Otago she has published over 100 research papers and received over $5M in research grant income for her work in improving clinical outcomes. Lisa is an Associate Editor for the International Journal of Nursing Studies and Chair of the national policy chapter on chronic disease with the Australian College of Nursing. Lisa will provide overall support and mentorship on this project.

Dr Travis Cruickshank is a Post-Doctoral Researcher in the MSWA research team and the director of the Huntington's disease research team at Edith Cowan University. Dr Cruickshank has been involved in Huntington’s disease research since 2010 and therefore has almost a decade of experience conducting research in this area. Dr Cruickshank has managed numerous successful trials assessing the effects of lifestyle interventions encompassing exercise, cognitive training, socialisation and sleep hygiene in individuals with Huntington’s disease. Dr Cruickshank has published numerous in the field of Huntington’s disease and has experience in delivering non-pharmaceutical interventions and assessing cognitive and motor function and lifestyle outcomes in individuals with neurological disorders.

Dr Johnny Lo is a Senior Lecturer in the School of Science. His area of expertise lies in applied statistics. Dr Johnny Lo has extensive experience as a statistician and has been involved in a number of industry projects, including those with Roc Oil Company Limited, Water Corporation WA, Woodside Energy, Rural and Remote Mental Health, and more recently, Department of Primary Industries and Regional Development. Most of his recent collaborations are in health domain; including mental health, nutrition, drugs, Huntington’s disease and melanoma studies. He has been involved in a number of successful grants, including a NHMRC grant in 2017. He brings a different set of skills to all the other collaborators in this tender and plays an important role as a statistician to ensure that there is rigour, integrity and credibility associated with the research methodology, analyses and findings and ultimately, decisions and recommendations arising from the work.

Dr. Jun Wen is a lecturer in tourism and hospitality management at the School of Business and Law, Edith Cowan University (ECU), Australia. Jun is currently devoted to interdisciplinary research focusing on mental health, public health, and tourism. Jun has also involved in a couple of projects examining the role of Traditional Chinese Medicine in Chinese inbound tourism market in the context of medical tourism. Relevant research has been published in internationally recognized journals such as Anatolia.

Dr Jianhong Cecilia Xia is an Associate Professor in the School of Earth and Planetary Sciences, Curtin University. Her area of expertise lies in human mobility studies. Dr Xia has published over 100 peer-reviewed journal and conference papers and book chapters. She has also been awarded over a million dollars in research grants from government agencies, industries and the Australian Research Council since 2005. She has many years’ experience in designing the survey and analysing the results.

# Research Scope, Aims, Themes, Questions and Methods

**Aims or Questions**

The overarching aim of this study is to provide compelling evidence of the feasibility and therapeutic efficacy of (low-level laser acupuncture LLLA) for office workers with shoulder and neck pain (SNP).

**Benefit of exploring these research questions**

Several studies demonstrated that LLLA is a promising modality in pain management. However, there is no consolidated evidence for LLLA in managing shoulders and neck pain, and more studies in this field are required to provide updated evidence. Therefore, this study aims to address this knowledge gap. We anticipate, based on past research, the LLLA we will provide may be helpful in reducing levels of SNP. We hope that the results of our research can be used to inform our knowledge about how to better manage SNP that many office workers are suffering. There are no foreseeable risks associated with this research project.

**Outline the design of this research; its methods and details of the instruments to be used to collect the data, including psychometric properties if applicable.**

**Study design:**

This is a single-blind (assessors blind to group allocation), double-armed, randomised controlled trial on the feasibility and therapeutic efficacy of a two-week LLLA therapy for office workers with SNP.

**Study type:**

Interventional study

**PICOs:**

P: Office worker aged 18-65, working more than 28 h a week with sedentary office work and have moderate to severe SNP (VAS > 5/10 & SF-MPQ >2/45) for more than three months will be recruited for this trial

I: LLLA + usual care

C: Usual care

O:

The primary objective of this study is to evaluate the feasibility of two-week LLLA for office workers with SNP.

The secondary objective is to evaluate the therapeutic efficacy of LLLA on pain, work productivity and activity, and quality of life in office workers with SNP.

S: This is a single-blind (assessors blind to group allocation), double-armed, randomised controlled trial on the feasibility and therapeutic efficacy of a two-week LLLA therapy for office workers with SNP.

**Intervention and exposure:**

The study will involve providing participants with LLLA to managing SNP.

All potential participants will be provided with an online information sheet and consent form outlining what will be involved in the trial. People who gave consent will complete the online survey after their signed the consent form.

 To reduce certain sources of bias when testing the efficacy of LLLA; this is accomplished by randomly allocating participants to two groups, one group that provides treatment and the other without treatment, and then comparing them with respect to a measured response. This is to provide the most reliable evidence on the efficacy of interventions because the processes used during an RCT minimise the risk of confounding factors influencing the results.

Participants will then be randomised to one of the two groups.

**Intervention group:**

In addition to usual care, participants will receive LLLA from a licensed acupuncturist at ECU Acupuncture Research Clinic. 10-20 minutes per session, three times a week for two weeks. The parameters of 3B Laser Pen (200mW) used for the intervention are wavelength of 808 nm in continuous wave mode, each pressure point receives 20 seconds of energy (4J) with 5 minutes being the maximum treatment time (60 J). At the end of two weeks receiving the therapy, participants will receive an online link to complete the post-intervention questionnaires.

**Control group:**

Non-intervention but usual care

Participants in this group will be informed via email that they are in the no treatment group but will be emailed an online link to complete the post-intervention questionnaire in two weeks’ time.

Participates in the non intervention group will receive a complementary LLLA after the two weeks trial.

**How the planned methods achieve the aim or research questions**

**Primary outcomes:**

The primary outcome for this study is feasibility of the two weeks LLLA therapy for office workers with SNP. Feasibility will be assessed by measuring: 1) recruitment and completion rates (No. of referred, No. of eligible, No. of enrolled, No. of withdrawals, trial recruitment rate, and trial completion rate), 2) patient safety (No. and severity of adverse events), and 3) treatment adherence (No. of completed sessions and missed sessions). Participants’ motivation and challenges to participation, withdrawal, missed sessions and non-compliance to intervention will be investigated, as will their attitude, and experience in participating the trial.

**Secondary outcomes:**

The questionnaires used in this project to assess the outcomes including demographics data, VAS, SF-MPQ, WPAI:SHP, SF-12, and the participants’ motivation, challenges, and attitude of participating the trial. As a feasibility study, questions on non-pharmacologic therapy and out-of-pocket (OOP) expenses will also be included.

**Statistical analysis:**

For the primary outcome, rates of recruitment (numbers consented/eligible), completion (undertaken baseline and follow-up tests), adherence (participant completed sessions/number of sessions), and adverse events (number and number per participant hour) will be calculated. Secondary outcome will be assessed following intention-to-treat principles. Repeated measures ANOVA will be conducted to assess changes in secondary outcomes throughout the study. This model allows inclusion of missing data in an intention-to-treat analysis without imputations (e.g. last-observation-carried-forward). If necessary, analysis will be adjusted for baseline levels and potential confounding factors. Normality assumptions will be assessed using the Shapiro-Wilk test. Statistical significance will be set at an alpha level of 0.05. Corrections will be applied to all analysed outcomes to account for multiple comparisons.

**Recruitment**

**Study population**

Although it is feasible to consist of 30 participants per group (Hertzog, 2008), some dropout is likely during the entire trial process. We estimate an attrition of 15% based on the attrition of 12% reported by previous study. Taking these two factors into account the sample size for this study is determined to be 70, with 35 participants in each of the two study groups.

Seventy Office worker aged 18-65, working more than 28 h a week with sedentary office work and have moderate to severe SNP (VAS > 5/10 & SF-MPQ >2/45) for more than three months will be recruited for this trial.

**Inclusion criteria**

1) aged between 18-65 years

2) working more than 28 h a week with sedentary office work

3) moderate to severe SNP (VAS > 5/10 & SF-MPQ > 2/45) for more than three months

**Exclusion Criteria**

1) with a serious health conditions of the neck and shoulder

2) pregnant

3) with cancer

4) high sensitivity to light

**How will participants be identified and initially contacted including screening processes?**

The snowballing process and public advertisement can identify potential participants. People interested in participating in the study will be encouraged to contact the research team via email for an eligibility check. We will send online screening checklist, including the SF-MPQ and VAS, to assess their suitability for participation.

**How will formal recruitment of potential participants be conducted?**

Our research team will contact those eligible to participate in the study and be the first 70 eligible protentional participants (first come, first serve) with a participant information letter and a link (starting with a consent form) to complete an online survey once they signed the consent form. The online survey should take no longer than 20 minutes to complete. Following the completion of the survey, the participants will be randomly allocated to one of the two groups

1. receive six laser acupuncture treatment sessions from a licensed acupuncturist at ECU Acupuncture Research Clinic.

2. receive NO treatment but usual care over two weeks’ trial time

Each treatment session lasts 10-20 minutes, three times a week for two weeks. At the end of two weeks receiving the therapy, participants will receive an online link to complete the post-intervention questionnaire.

Participants in the Non treatment group will receive a complementary laser acupuncture treatment after the two weeks trial.

The recruitment process will be taking place in the public, and the 10-20 minute sessions occur outside working hours. For these reasons, approval from employers is not required.

**Please provide a response to any ethical issues and concerns raised by the recruitment process such as the relationship between researchers and participants, risks associated with recruitment strategy, and the nature of the population being examined.**

There is no single correct approach, but a balanced recruitment approach will be employed (e.g., use snowballing as well as public advertisement). Recruitment of participants should be viewed as part of the research protocol and should require appropriate informed consent of the already-enrolled participant. Investigators should inform prospective participants why they are being contacted, how information about them was obtained, and what will happen to that information if they decide not to participate.

**Data collection method and location including who will be responsible for the data collection**

Participation in this research project is voluntary. If one does not wish to take part, they do not have to. If participant decided to take part and later changed their mind, they are free to withdraw from the project at any time. If they decide to withdraw from the project after the data has been analysed, we will not be able to remove the individual data as this cannot be identified.

If participant do decide to take part, they will be given the participant information letter and consent form to sign, and they will be given a copy of the information letter to keep. They decision to take part, or to take part and later withdraw, will not affect they relationship with the research team and any staff within the School of Nursing and Midwifery at ECU.

By signing the consent form, they consent to the research team collecting and using personal information about the participant for the research project. Any information obtained in connection with this research project that can identify participant will remain confidential. When all survey responses are returned to the research team, all data are automatically de-identified, and they will not be identifiable by any of their responses to the survey. They information will only be used for the purpose of this research project and it will only be disclosed with they permission, except as required by law.

It is anticipated that the results of this research project will be published and/or presented in a variety of professional forums. In any publication and/or presentation, the information will be provided in such a way that participants cannot be identified, except where requested for specific reasons, and then they will be asked to provide written consent.

In accordance with relevant Australian and/or Western Australian privacy and other relevant laws, participants have the right to request access to the information about them that is collected and stored by the research team. They also have the right to request that any information that they disagree to be corrected. They are encouraged to inform the research team member named at the end of this letter if they would like to access they information.

All data collected will be kept in accordance with ECU’s Data Management Policy. Electronic data will be stored on a secure Microsoft SharePoint site provisioned by ECU’s IT Services and physical records will be stored as required in ECU’s Records Management Policy. The data will be retained for a period of seven years and destroyed, if appropriate at the end of the retention period. Data will be de-identified when stored and at the end of the retention period, the data will be destroyed, if appropriate under the State Records Act.

There are no foreseeable risks associated with participation in this research project.

We will advise participants of the outcomes via email communication. We also intend to publish our results in research journals and present them at research conferences locally, nationally and internationally. Participants' name or any other identifying information will not be included in any of the publications or presentations.

This research project will not start until we sought the approval of Edith Cowan University’s Human Research Ethics Committee under the National Health and Medical Research Council’s National Statement on Ethical Conduct in Human Research 2007 (Updated 2018).

**Statistical analysis**

For the primary outcome, rates of recruitment (numbers consented/eligible), completion (undertaken baseline and follow-up tests), adherence (participant completed sessions/number of sessions), and adverse events (number and number per participant hour) will be calculated. Secondary outcome will be assessed following intention-to-treat principles. Repeated measures ANOVA will be conducted to assess changes in secondary outcomes throughout the study. This model allows inclusion of missing data in an intention-to-treat analysis without imputations (e.g. last-observation-carried-forward). If necessary, analysis will be adjusted for baseline levels and potential confounding factors. Normality assumptions will be assessed using the Shapiro-Wilk test. Statistical significance will be set at an alpha level of 0.05. Corrections will be applied to all analysed outcomes to account for multiple comparisons.

**Risk**

**Physical Risks**

LLLA is cleared and approved by the Food and Drug Administration (FDA). It is safe and there are no foreseeable risks associated with participation in this research project. However, both the practitioner and the participants will wear an appropriate laser safety eyewear that match the laser wavelength and have sufficient optical density at that wavelength to protect the eye.

**Psychological Risks**

Participants in the Non treatment group may feel disappointed. They will receive a complementary LLLA treatment after the two weeks trial as our appreciation and acknowledgement of their time.

**Social Risks**

No foreseeable social risks associated with participation in this research project.

**Participant Information Letter**

**Project title:** The efficacy of laser acupuncture for shoulder and neck pain in office workers: A double blinded, placebo controlled randomised trial

**Approval Number:** 2021-02225-WANG

**Trial registration No.** ACTRN12621000426886p

**Principal Investigator:** Dr Carol Wang

**An invitation to participate in research**

You are invited to participate in a project titled **“The feasibility and therapeutic efficacy of a two-week low-level laser acupuncture therapy for shoulder and neck pain in office workers: a single-blind, double-armed, randomised controlled trial***”.* You are being asked to take part in this project because you are aged between18 and 65 years, working more than 28 h a week with sedentary office work, with shoulder and neck pain for more than 3 months.

Please read this information carefully. Ask questions about anything that you do not understand or want to know more about. Before deciding whether to take part, you might want to talk about it with a relative or friend.

If you decide you want to take part in the research project, you will be asked to sign a consent form. By signing it, you are telling us that you:

- Understand what you have read;
- Consent to take part in the research project;
- Consent to be involved in the research described;
- Consent to the use of your personal information as described.

**What is this project about?**

Shoulder and neck pain (SNP) are common and burdensome conditions in office workers with 42-63% prevalence. It is a serious public health problem and has a significant detrimental impact on one’s quality of life, physical functioning, economic structure, families, employers, and healthcare system.

Lengthened working hours on the computer, prolonged sitting position, and static postures are the most contributing factors, and medication and physiotherapy were the most common intervention strategies that people chose to manage their pain.

The low-level laser therapy (LLLT), also known as cold laser, photobiomodulation, applies specific wavelengths and low power density, has been shown to be an additional option in the SNP management to relieve pain and re-establish quality of life. LLLT can promotes analgesic and anti-inflammatory effects, improve blood circulation, boosts immunity and speeds up wound healing, with no reported adverse effects and well received by many patients.

Low-level laser acupuncture (LLLA), is one of the recent technological developments (e.g., electroacupuncture) in the practice of acupuncture, which integrate the cutting-edge laser technology and thousands of years old TCM modality. Instead of the needle stimulation on the acupuncture points, low intensity non thermal laser irradiation is applied to elicit the cellular level’s physiologic effects with sufficient energy. It is, therefore, non-invasive, painless, non-infectious, and safe to use. LLLA has become increasingly attractive for patients with needle phobias, older people and children.

Several studies demonstrated that LLLA is a promising modality in pain management. However, there is no consolidated evidence for LLLA in managing shoulders and neck pain, and more studies in this field are required to provide updated evidence. Therefore, this study aims to investigate the feasibility and therapeutic efficacy of LLLA for office workers with SNP.

**Who are the people should not participate in this project?**

Women who are pregnant, people with cancer, or high sensitivity to light will not be eligible.

**What does my participation involve?**

Your participation in this research project will involve completing an online survey which will require you to sign a consent form, completing a set of questionnaires including demographics questions and questions to measure pain levels, work productivity and activity, quality of life, and information on the use of nonpharmacologic therapies for your shoulder and neck pain. The online survey should take you no longer than 20 minutes to complete. Following the completion of the survey, you will be randomly allocated to one of the two groups:

1. receive six laser acupuncture therapy sessions from a licensed acupuncturist at ECU Acupuncture Research Clinic

Or

1. receive NO treatment over the two-week trial time

You will then receive an online link to complete the post-intervention questionnaire at the end of two-week trial time.

For the laser acupuncture therapy, each session lasts 10-20 minutes, three times a week for two weeks.

If you are in the Non treatment group, you will receive a complementary laser acupuncture therapy after the two weeks trial.

**Do I have to take part in this research project?**

Your participation in this research project is voluntary. If you do not wish to take part, you do not have to. If you decide to take part and later change your mind, you are free to withdraw from the project at any time. If you decide to withdraw from the project after the data has been analysed, we will not be able to remove your individual data as this cannot be identified.

If you do decide to take part, you will be given a consent form to sign, and you will be given a copy of this information letter to keep. Your decision to take part, or to take part and later withdraw, will not affect your relationship with the research team and any staff within the School of Nursing and Midwifery at ECU.

**Your privacy**

By signing the consent form, you consent to the research team collecting and using personal information about you for the research project. Any information obtained in connection with this research project that can identify you will remain confidential. When all survey responses are returned to the research team, all data are automatically de-identified, and you will not be identifiable by any of your responses to the survey. Your information will only be used for the purpose of this research project and it will only be disclosed with your permission, except as required by law.

It is anticipated that the results of this research project will be published and/or presented in a variety of professional forums. In any publication and/or presentation, the information will be provided in such a way that you cannot be identified, except where requested for specific reasons, and then you will be asked to provide written consent.

In accordance with relevant Australian and/or Western Australian privacy and other relevant laws, you have the right to request access to the information about you that is collected and stored by the research team. You also have the right to request that any information that you disagree to be corrected. Please inform the research team member named at the end of this letter if you would like to access your information.

All data collected will be kept in accordance with ECU’s Data Management Policy. Electronic data will be stored on a secure Microsoft SharePoint site provisioned by ECU’s IT Services and physical records will be stored as required in ECU’s Records Management Policy. The data will be retained for a period of seven years and destroyed, if appropriate at the end of the retention period. Data will be de-identified when stored and at the end of the retention period, the data will be destroyed, if appropriate under the State Records Act.

**Possible Benefits**

We anticipate, based on past research, that laser acupuncture treatment that we will provide may be helpful in reducing levels of shoulder and neck pain, increasing work productivity and quality of life. We hope that the results of our research can be used to inform our knowledge about how to better manage shoulder and neck pain that many office workers experience.

There are no foreseeable risks associated with your participation in this research project.

**Possible Risks and Risk Management Plan**

There are no known risks to participating in this research project.

**What happens when this research study stops?**

We will advise you of the outcomes via email communication. We also intend to publish our results in research journals and present them at research conferences locally, nationally and internationally. Your name or any other identifying information will not be included in any of the publications or presentations.

**Has this research been approved?**

This research project has received the approval of Edith Cowan University’s Human Research Ethics Committee under the National Health and Medical Research Council’s *National Statement on Ethical Conduct in Human Research 2007 (Updated 2018)*. The approval number is 2021-02225-WANG.

**Contacts**

If you would like to discuss any aspect of this project, please contact the following people.

| **Chief Investigator** |  |
| --- | --- |
| Dr Carol Wang |  |
| Teaching and research academic |  |
| Edith Cowan University |  |
| P: 6304 3589 |  |
| E: c.wang@ecu.edu.au |  |

If you have any concerns or complaints about the research project and wish to talk to an independent person, you may contact:

| **Independent Person** |
| --- |
| Research Ethics Support Officer |
| Edith Cowan University |
| P: 6304 2170 |
| E: research.ethics@ecu.edu.au |

**Participant Consent Form**

**Project title:** Laser acupuncture for shoulder and neck pain in office workers: A single blinded randomised controlled feasibility trial

**Approval Number:** 2021-02225-WANG

**Trial registration No.** ACTRN12621000426886p

**Principal Investigator:** Dr Carol Wang

I, __________________________________ have read the Participant Information Letter. By signing this consent form, I acknowledge that I:

- have been provided with a copy of the Participant Information Letter, explaining the research study
- have read and understood the information provided
- have been given the opportunity to ask questions and have had questions answered to my satisfaction
- can contact the research team if I have any additional questions
- understand that participation in the research project will involve:
  - completion of an online survey,
  - assigned into either a non-treatment group or treatment group receiving six sessions of laser acupuncture therapy in two weeks from a licensed acupuncturist at ECU Acupuncture Research Clinic, and
  - completion of an online survey which is the post-intervention questionnaire.
- understand that the information provided will be kept confidential and that my identity will not be disclosed without consent
- understand that I am free to withdraw from further participation at any time, without explanation or penalty
- freely agree to participate in the project.
- The data collected will be used only for this research project.

| Participant name: |  | | |
| --- | --- | --- | --- |
| Signature: |  | Date |  |
